# Integrated monitoring of training and sport performance throughout an entire handball season: practical applications in semi-professional female players

**DOI:** 10.3389/fspor.2026.1869707

**Published:** 2026-06-24

**Authors:** Antanas Skarbalius

**Affiliations:** Department of Coaching Science, Lithuanian Sports University, Kaunas, Lithuania

**Keywords:** A:CWR, match performance, neuromuscular fatigue, retraining and competition microcycles, sRPE, strength and endurance fitness, well-being

## Abstract

**Background:**

The aims of this study were 1) to investigate the seasonal dynamics of training load, neuromuscular status, athlete mental state, and physical performance indicators and 2) to determine how these variables and training content relate to match performance and outcomes in semi-professional female handball players. Additionally, this study examined the practical application of monitoring-informed periodization using a structured microcycle model.

**Methods:**

A longitudinal observational study was conducted across the entire competitive season (193 days, 159 training sessions, 34 matches) of semi-professional female handball players. The internal training load was quantified using the session rating of perceived exertion multiplied by the session duration (min), allowing the calculation of the total weekly load, monotony, strain, and acute:chronic workload ratio. Neuromuscular status was assessed using the countermovement jump (CMJ), whereas the athlete’s mental state was evaluated using a five-component well-being questionnaire. Physical performance indicators (strength and intermittent endurance) and sport-specific performance (throwing velocity) were also assessed. The match performance was analyzed using notational analysis. The training content was categorized into physical, technical, tactical, integral, recovery, warm-up, and theoretical components.

**Results:**

Major performance findings demonstrated significant improvements in intermittent endurance and strength endurance, whereas CMJ remained unchanged throughout the season. The internal load varied systematically across microcycles, with higher monotony and strain during the competitive phases. Match outcome was primarily associated with defensive performance variables, particularly first-half defensive efficiency (r_pb = 0.680, *p* = 0.011) and goalkeeper effectiveness (r_pb = 0.576, *p* = 0.039). Acute training content, especially tactical (*r* = 0.54) and integral training (*r* = 0.48), demonstrated the strongest relationship with match outcome.

**Conclusion:**

Performance in semi-professional female handball players is best understood as an integrated system in which the training load (including total weekly load, monotony, strain, and acute:chronic workload ratio), athlete well-being, and tactical preparation interact dynamically. Match success was primarily associated with defensive efficiency and short-term sport-specific preparation. These findings support monitoring-informed, periodization approach based on a structured microcycle model (retraining, one match, and two match competition microcycles), demonstrating that effective performance development can be achieved without a traditional long preparatory phase when training loads are coherently structured throughout the seasons.

## 1 Introduction

In team handball, as in other sports (1), player performance is strongly influenced by the training process aimed at improving sport-specific fitness, technical proficiency, tactical effectiveness, and readiness for competition. However, performance development in female handball cannot be explained solely through physiological adaptation to training loads. Contemporary coaching theory increasingly emphasizes that training effectiveness also depends on the extent to which the coaching philosophy and periodization model are aligned with the specific characteristics of the athlete group, competitive environment, and contextual constraints (2, 3). In semi-professional female handball, these constraints often include limited training time, congested competition schedules, academic or occupational commitments, and restricted recovery opportunities. Consequently, individualized and context-sensitive coaching approaches may be equally or even more effective than rigid traditional training models when they are coherently integrated with the needs of the target population.

Sport performance was determined based on the content of the training program. Buchheit (4) states that successful handball performance depends 45% on technical, 35% on physical, and 20% on tactical preparedness. However, when training female handball players, part of the training time must be allocated for warm-up and recovery (5). Skarbalius et al. (5) identified a seven-element distribution structure of training content through long-term studies: warm-ups, 22.2%; recovery, 11.3%; integral training, 25.1%; physical training, 15.8%; technical training, 12.5%; tactical training, 9.8%; and theoretical training, 3.3%. Integral training (5) refers to training situations in which all components of preparedness are developed simultaneously. This category includes official and friendly matches, as well as other forms of sport-specific practice (for example, small-sided games), where technical, tactical, physical, and cognitive elements are integrated within a unified performance context. A key problem arises as to how such a structure could and should be applied across different microcycles when training semi-professional female handball players throughout the season.

The volume of applied load is a particularly important factor in training handball players (6). The available evidence indicates that female handball players typically accumulate approximately 460 min per week of exposure in youth elite settings (7), that a single training session may generate approximately 380 AU of internal load (8), that a competitive match commonly elicits ≥ 630 AU in elite adult women (9), and weekly accumulated internal load values of approximately 2,470 AU have been documented in season-long monitoring of female players (7). However, published data describing the full-season weekly session rating of perceived exertion (sRPE) of microcycles throughout the season in semi-professional women’s handball remain limited, highlighting the need for longitudinal monitoring studies in this population.

The recent increase in competition density and extension of the competitive calendar have intensified the debate on the applicability of traditional periodization models throughout the sports season (10). In semi-professional female handball, where training time is constrained, recovery resources are limited, and match density is high, rigid preplanned periodization structures may lack ecological validity. Under these conditions, season planning should shift from a model-driven approach to a monitoring-informed, adaptive framework in which weekly and microcycle adjustments are guided by objective and subjective load indicators rather than fixed phase structures (5). Periodization should function as a dynamic organizational strategy, continuously adjusted according to performance and fatigue indicators, rather than as a predetermined sequence of fixed phases (10). Although Manchado et al. (11) concluded that block periodization may be more effective than traditional approaches for improving key physical and physiological performance factors in elite female handball players, this evidence supports a seasonal strategy in which short, concentrated training blocks (e.g., emphasizing strength–power development) are strategically implemented at selected time points, while the remaining weekly structure prioritizes match readiness and fatigue management. Owing to the inevitable biologically driven processes of detraining, traditional periodization models may lose both practical coherence and functional relevance during prolonged competitive phases (12, 13). Consequently, the primary tool for managing sport performance in female handball players should be structured programming of competition microcycles (9) guided by continuous performance and load monitoring (14). Between competitive microcycles, targeted retraining microcycles should be implemented to restore and reinforce performance capacities that may decline during periods with a high number of matches. Retraining responses are typically faster than initial training adaptations because of retained neuromuscular and structural memory; however, load progression must be carefully controlled to minimize fatigue and injury risk during retraining (14–16). Microdosing, defined as the frequent administration of small high-quality training stimuli, is an acceptable, physiologically supported method for retraining, particularly when the goal is to restore neuromuscular performance without excessive fatigue accumulation (17). In female handball players, a short-term (approximately 3-week) detraining period significantly impairs neuromuscular performance, particularly explosive lower-limb function, emphasizing the need for carefully structured retraining or maintenance strategies during season interruptions (18). Consequently, the practical value of microdosing as a retraining or maintenance strategy in a semi-professional female handball population remains unclear because of the absence of sport- and population-specific empirical evidence defining its optimal dosing parameters, effectiveness, and interaction with competition-induced fatigue.

A recent athlete monitoring survey in handball (ATHMON-HB) of professional women and men reported how teams currently implement athlete monitoring systems and which internal and external measures were most common (14). Semi-professional female handball presents a complex performance environment characterized by congested competition schedules, dual career demands, and limited recovery resources (19). Under these constraints, integrated and feasible monitoring systems are essential for managing performance and fatigue. Internal load quantification using rating of perceived exertion (RPE) and sRPE remains a practical and validated approach in team sports (20). The validity of sRPE as an internal load indicator in female handball players has been supported by correlational evidence. Findings from female handball players have confirmed that sRPE-derived volume reflects both mechanical and physiological stress (21). However, weekly load totals alone may fail to capture harmful load distributions. Therefore, indices such as monotony, total weekly load (TWL), and strain have been introduced to quantify the variability and accumulated stress across microcycles (22, 23). Similarly, the acute:chronic workload ratio (A:CWR) has been proposed to detect rapid load spikes associated with injury risk (15), although its methodological limitations require cautious interpretation (24). Load monitoring must be complemented by athlete mental state surveys. A systematic review of evidence demonstrated that well-being questionnaires are sensitive to training stress and may detect maladaptation earlier than objective measures (25). In parallel, countermovement jump (CMJ) monitoring provides a practical indicator of neuromuscular readiness and fatigue status (26, 27). In addition to load and readiness, season management in female handball players requires monitoring of strength, endurance capacity, and body composition. Seasonal fluctuations in aerobic fitness and strength have been documented in elite female handball players, demonstrating that competitive phases may stabilize or reduce certain physical capacities if not maintained systematically (28). Endurance performance markers, such as the Yo-Yo test, are sensitive to both detraining and retraining processes in team sport athletes (29), underscoring the importance of periodic assessment. Additionally, changes in body composition across competitive seasons can influence performance and injury risk profiles, particularly in female athletes (28). Finally, performance validation requires linkage to notational analysis, as match-derived indicators (e.g., defensive and offensive efficiency, fast breaks, shooting efficiency, turnovers, and goalkeeper effectiveness) differentiate winning and losing teams in handball (30–32). Despite the availability of these monitoring tools, there is limited empirical guidance on how to integrate internal load indices, neuromuscular readiness, strength and endurance monitoring, body composition, and match analytics into a coherent decision-making framework for semi-professional female handball.

In team handball, increasing match congestion, extended competitive calendars, and the multidimensional nature of performance require more context-sensitive approaches to training management, particularly in semi-professional settings. In semi-professional female handball, training processes are further constrained by limited training time and restricted recovery resources, which may reduce the applicability of rigid preplanned periodization models. Consequently, ecologically valid integrated monitoring approaches that capture the dynamic interactions among training load, athlete responses, and match performance across the entire competitive season are needed. Although previous studies have examined isolated components such as internal load, neuromuscular fatigue, and subjective well-being, few studies have investigated these variables within a comprehensive, season-long framework in semi-professional team sports contexts.

The aims of this study were: 1) to investigate the temporal dynamics and interrelationships among key training and performance variables across the competition season, and 2) to establish reference ranges for integrated monitoring variables to support adaptive, data-informed microcycle adjustments in semi-professional female handball.

## 2 Materials and methods

### 2.1 Research design

This observational study was conducted across the entire 2021–2022 handball season, spanning nine months. The team began training on August 23, 2021, and ended the competitive season on May 29, 2022; thus, the observation period lasted 193 days of training and competition. During this period, players completed 159 training sessions and participated in 34 matches, including 26 official competitions. A typical weekly structure consisted of four training sessions and one match. However, during the final competitive phase, match congestion increased and the selected microcycles included two matches per week. Across the season, 26 official matches were distributed across four competitive formats: the EHF European Cup (two matches), Lithuanian Championship (21 matches), Lithuanian Cup (two matches), and Lithuanian Supercup (one match). Additionally, eight friendly matches were played. All training sessions and matches were continuously monitored throughout the season. This study was designed to provide ecologically valid insights into training load, fatigue dynamics, and performance management under real-world conditions in semiprofessional female handball players. Monitoring indicators (Table 1) were collected before, during, and after the sessions to assess the athletes’ responses to loads, microcycle fluctuations, changes in body composition, and longitudinal adaptations in strength and endurance. The performance indicators for all matches were recorded, and each player was provided with their individual analysis.

**Table 1:** Monitoring design.

Before session	During session	After session	Microcycle (weekly)	Changes throughout the season
Subjective well-being questionnaire Mental state (fatigue, sleep quality, muscle soreness, stress, mood, total well-being) Neuromuscular fatigue (countermovement jump)	Training and match-play content (training duration in minutes and % distribution) Match notational analysis	Subjective load RPE sRPE	Monday 6:00 pm Body composition Monotony Training weekly load Strain A:CWR	Different months Throw velocity Dynamic and isometric strength Endurance

A:CWR, acute:chronic workload ratio; RPE, rating of perceived exertion; sRPE, session rating of perceived exertion.

### 2.2 Participants

Fourteen semi-professional female handball field players (aged 25.1 ± 6.0 years; national-level playing experience, 4.3 ± 3.4 years; height, 172.8 ± 6.2 cm; body mass, 67.9 ± 6.3 kg) participated in this longitudinal observational study. All the players were members of the same team and competed in the highest national league. During the 2021–2022 season, the team won both the Lithuanian Championship, Lithuanian Cup, and Lithuanian Super Cup. Players were classified as semi-professionals because they combined structured team training and official competition with occupational or academic commitment. All 14 players were included in the monitoring process. Owing to minor injuries, illnesses, or match-related constraints, the number of players available for individual testing sessions varied; however, each testing session included data from at least 12 players. Data were collected under real-world team conditions, using a repeated-measures observational design. All participants were informed of the study procedures and provided written informed consent prior to participation. This study was conducted in accordance with the Declaration of Helsinki and approved by the Kaunas Regional Biomedical Research Ethics Committee (reference number: BE-2-55).

### 2.3 External load assessment

The external load was assessed on the basis of the volume and content of the training load. The training content was recorded in accordance with the methodology used in long-term monitoring studies (5). Based on a structured recording protocol (spreadsheet), the assistant coach recorded the duration (in minutes) of each training session for warm-up, integral training, physical training, technical training, tactical training, theoretical training, and recovery. Inter-observer agreement was established prior to data collection. The percentage distribution of each content element and total duration of each training session were calculated. The same spreadsheet was used to calculate the microcycle training loads (in min) and the percentage distribution of the training content across microcycles.

### 2.4 Internal load assessment

The internal training load was quantified using the rating of perceived exertion (RPE) method and its session-integrated form (sRPE) according to the procedure described by Foster et al., and the reported RPE values were multiplied by the training duration to obtain the s-RPE (20). The RPE and sRPE are practical and valid methods for assessing internal load across a variety of team sports and have been successfully applied in studies of female handball players (5, 8, 9, 33). At the end of each training session and match, the players were asked to report their overall perception of exertion using the Borg CR-10 scale. To ensure that the reported value reflected the cumulative stress of the entire session rather than the final exercise set, the RPE was collected 20–30 min after session completion. Players were instructed to indicate a single RPE value representing the global intensity of the session (“How hard was your session?”). Players had systematically recorded RPEs over several years and developed the ability to adequately and reliably assess the intensity of their exertion. To characterize the weekly load distribution, monotony and strain were calculated (22). A spreadsheet-based algorithm was developed to automatically compute the monotony, weekly training load, and strain.

Monotony=meandailyload/standarddeviationofdailyload

TWL=sumofallthesessionloads

Strain=TWL×monotony

### 2.5 A:CWR assessment

Despite its limitations as an injury prediction metric, the A:CWR may still serve as a practical tool for guiding training load management and supporting decision-making within the athlete preparation process when interpreted within a broader monitoring framework (34–36). The A:CWR was calculated to quantify week-to-week load fluctuations, where acute load represented the current week’s total sRPE and chronic load was defined as the rolling average of the previous four weeks (37). Gabbett et al. (38) compared coupled and uncoupled formulations of the A:CWR and reported an almost perfect agreement between the two approaches, with no meaningful differences; therefore, in the present study, the coupled method was adopted.

### 2.6 Well-being

Subjective self-reported measures of well-being such as fatigue, sleep quality, muscle soreness, and stress have demonstrated valid, reliable, and sensitive associations with training load and recovery status in athletes, making them effective tools for monitoring internal load and perceptual fatigue responses in high-performance sports settings (25). The well-being of the team’s handball players was assessed using a questionnaire developed by McLean et al. (39), which was deemed to be practical and reliable despite its subjective nature. Prior to each training session, the players individually completed the five-item well-being scale (Table 2) and submitted their responses to the coach before performing the CMJ test. The composite well-being score was calculated as the sum of all five individual components. Based on the reported well-being status, the coach evaluated each athlete’s readiness, clarified potential underlying causes when necessary, and, through mutual agreement, adjusted the nature and content of the training sessions accordingly.

**Table 2:** Example well-being assessment form (37).

ŽALGIRIS player _________________________________						
Date: ____________________ Hour: _______________						
VALUES	5	4	3	2	1	Record score
FATIGUE	Very fresh	Fresh	Normal	More tired than normal	Always tired	
SLEEP QUALITY	Very restful	Good	Difficulty falling asleep	Restless sleep	Insomnia	
GENERAL MUSCLE SORENESS	Feeling great	Feeling good	Normal	Increase in soreness/tightness	Very sore	
STRESS LEVELS	Very relaxed	Relaxed	Normal	Feeling stressed	Highly stressed	
MOOD	Very positive mood	A generally good mood	Less interested in others &/or activities than usual	Snappiness at team-mates, family and co-workers	Highly annoyed/irritable/down	
Well-being (Total)						

### 2.7 Neuromuscular fatigue

CMJ performance is a valid and reliable field criterion for monitoring neuromuscular fatigue, showing good test–retest reproducibility and sensitivity to fatigue-induced changes in neuromuscular function, with meta-analytic and review evidence supporting its use for day-to-day readiness monitoring in high-performance team sport contexts (40, 41). CMJ was assessed as originally described by Bosco et al. (40). The Bosco jump methodology is based on the assessment of lower-limb neuromuscular performance through vertical jump tests that quantify explosive force production and stretch–shortening cycle efficiency. Jump height was calculated from flight time using the equation *h* *=* *g·t²/8*, where *g* represented gravitational acceleration (9.81 m·s⁻²), and *t* was the measured flight time obtained via a contact platform that detects take-off and landing through electrical circuit interruption (40). This methodology has demonstrated high reliability for vertical jump assessment and has been extensively applied in team sports to monitor neuromuscular status, fatigue, and readiness to perform (41, 42). Based on prior applications of the CMJ in studies involving elite female handball players (42), this test was selected as the primary criterion for assessing neuromuscular fatigue in teams. Neuromuscular performance was assessed before each training session or match play using the CMJ. The players performed a maximal vertical jump with a self-selected knee flexion depth during the countermovement phase and a free arm swing throughout the movement using the Ergojump–Bosco system platform (43). This execution protocol was chosen to enhance ecological validity, as it allows individualized knee angles and natural arm actions to more closely replicate the movement patterns and coordination demands encountered during match-play in handball. The handball players had been performing this jump protocol for several years (each completing several hundred trials) and therefore demonstrated highly developed and technically consistent jumping proficiency.

### 2.8 Body composition monitoring

Body composition monitoring is a critical component of athlete performance management in team sports as it provides objective information about fat and fat-free mass and their segmental distribution that directly influences force production, power-to-mass ratio, speed, and injury risk (44). Regular assessment enables practitioners to evaluate long-term training adaptations, individualize nutrition and strength training interventions, and contextualize performance test outcomes (e.g., CMJ) relative to changes in body mass composition rather than total body mass alone (45). From a physiological perspective, optimal lean mass supports neuromuscular performance, whereas excessive fat mass may negatively affect repeated sprint ability and vertical jump performance due to an increased inert load (45). Therefore, body composition monitoring contributes to both training programs and performance optimization, particularly during long competition periods (45). Body composition was assessed using a multi-frequency bioelectrical impedance analyzer (TANITA, Tokyo, Japan), a method that has been widely applied in sports science research to estimate body, fat, and lean body masses in team sport athletes (45). Measurements were performed under standardized conditions to enhance the reliability and minimize the hydration-related variability inherent in bioelectrical impedance analysis. Assessments were consistently conducted on Mondays, the first day of the microcycle at 18:00. Players were instructed to consume lunch at a standardized time between 14:00 and 15:00 to control for potential nutritional influences on the measurements.

### 2.9 Physical attributes

#### 2.9.1 Dynamic and isometric strength capabilities

Dynamic and isometric strength capacities are essential performance determinants in handball and resistance training significantly enhances maximal and isometric force production, which underpins jump, sprint, and throw performances (46–49), while dynamic strength development training improves functional performance in elite female players (47). McMaster et al. (49) highlighted that dynamic, ballistic, and isometric strength assessments provide complementary insights into neuromuscular performance, recommending integrated testing approaches that align with the force–velocity demands of sport-specific actions. Continuous monitoring of strength indicators enables the evaluation of the effectiveness of implemented strength training programs (48, 49). In semi-professional female handball players, the assessment of dynamic and isometric strength does not involve complex laboratory equipment; therefore, field-based tests were employed. At the beginning of each microcycle, three tests were performed on Mondays following a warm-up: 1) flexed-arm hang to assess isometric upper limb strength, 2) 30-second sit-up test to assess dynamic trunk strength, and 3) plank hold to assess isometric core strength.

#### 2.9.2 Endurance capabilities

Elite female handball is physiologically demanding, with players operating at high percentages of their maximal heart rate and performing repeated intermittent efforts. Michalsik et al. (50) reported that elite female players typically perform large numbers of accelerations and high-intensity runs during competition, highlighting the importance of aerobic fitness in maintaining the work rate throughout the match. Aerobic power (VO₂max) and intermittent endurance capacity have been shown to influence the recovery kinetics of repeated high-intensity actions. In a systematic review, Póvoas et al. (46) concluded that aerobic fitness is essential for sustaining repeated sprint performance and match intensity in elite handball. Research on elite female handball players has shown that insufficient aerobic conditioning may contribute to the accumulation of neuromuscular fatigue during congested competition schedules (46, 51). Ronglan et al. (42) demonstrated a significant decrease in neuromuscular performance during tournament performance, indirectly supporting the role of aerobic capacity in recovery efficiency. Aerobic conditioning in female handball players is not aimed at continuous steady-state endurance, but rather at improving:

- Repeated high-intensity effort capacity
- Recovery between sprints and contacts
- Maintenance of technical performance under fatigue
- Match-to-match recovery during congested schedules

High-intensity interval training and sport-specific intermittent conditioning have been recommended as effective strategies for enhancing aerobic fitness in team sports (52).

Maximal oxygen uptake (VO₂max) was determined at the Institute of Sport Science and Innovations of the Lithuanian Sports University using an incremental treadmill test with breath-by-breath gas exchange analysis via a calibrated metabolic cart. Assessments were conducted twice during the season: at the beginning of the season in October, and prior to the playoff phase in April. To evaluate changes in aerobic endurance, the Yo-Yo Intermittent Endurance Test Level 1 (YYIE1) was administered six times during the retraining periods (microcycles 1, 7, 14, 18, 23, 28, and 35). The YYIE1 Test was performed according to standardized procedures described in the original Yo-Yo testing manual (53), with the physiological rationale detailed in subsequent peer-reviewed publications (54). In addition to serving as an assessment tool, this testing procedure was also incorporated as a high-intensity endurance training stimulus aimed at eliciting maximal effort from players.

### 2.10 Throwing velocity

Throwing velocity has been shown to improve with strength-oriented interventions in female handball players (classic example: maximal strength training and strength–power), supporting its use as an outcome for evaluating training effectiveness (55–58). The ball throwing velocity was assessed using a calibrated Doppler radar gun (Stalker Pro, Applied Concepts Inc., USA; sampling frequency 100 Hz) positioned directly behind the goal and aligned with the trajectory of the ball to minimize angular measurement error. After a standardized sport-specific warm-up, players performed three maximal-effort throws under standardized conditions: a 7 m standing throw (penalty style), a 9 m three-step running throw, and a 9 m three-step jump throw. A 1–2 min passive recovery period was allowed between trials to minimize fatigue effects. The highest recorded ball velocity (km·h⁻¹) from the three attempts was retained for analysis, as this procedure has been shown to provide reliable and sensitive assessment of throwing performance in female handball players (57, 59). The assessments were conducted six times throughout the season during the same retraining microcycles as YYIE1 (microcycles 1, 7, 14, 18, 23, 28, and 35).

### 2.11 Notational analysis

Notational analysis in female handball refers to the systematic recording and quantification of technical–tactical performance indicators during matches to objectively describe performance structure, identify success determinants, and support training prescriptions (60). In elite female handball players, notational analyses have consistently shown that match success is strongly associated with shooting efficiency, goalkeeper save percentage, reduction in technical errors (turnovers), and effectiveness in fast break situations (60). Although notational analysis in handball has been extensively investigated, clear and universally accepted game-related performance criteria capable of reliably predicting match outcomes have not been established. The discriminatory power of specific indicators appears to vary depending on the competitive level, with certain variables exerting greater influence in one context and other variables becoming more decisive in another (30–32). Nevertheless, the systematic and longitudinal application of notational analysis within a team provides a structured basis for shaping and refining the team’s playing style in accordance with the players’ skill levels and performance characteristics. To date, benchmark values for competitive performance indicators in semi-professional female teams have not been established. It remains unclear whether these indicators correspond to elite-level standards or differ substantially from those observed in top-tier competition. Throughout the competitive season, match performances were systematically recorded across all 34 official games. Team actions were analyzed according to the following criteria: number and effectiveness of defensive and offensive attacks, number and effectiveness of positional attacks and fast breaks, number and efficiency of shots from different court positions, number and effectiveness of majority and minority attacks, positive actions (e.g., steals, breakthroughs, earned 7 m penalties, and blocked shots), negative actions (e.g., 2 min suspensions and turnovers), and goalkeepers’ total saves and save efficiency. Individual player actions were recorded and analyzed using the same structured framework. Match performance was registered by an assistant coach. The validity of the action recording was verified through a comparison with the official match report, whereas shot data were cross-checked against an independent shot-registration protocol completed by the substitute goalkeeper. When necessary, all actions were further verified through detailed video analysis of the matches. Due to differences in team performance levels during the Lithuanian Championship, only matches with a final goal difference of fewer than seven goals were included in the analytical dataset (*n* = 13). Directly after each match, the head coach conducted a comprehensive team and individual performance analysis, which was distributed later to each player. Additionally, qualitative individual video analyses of all the matches were performed using the Stat4Sport platform. After particularly important matches, to evaluate performance quality and to prepare and model upcoming game strategies, all players reviewed and analyzed match footage together with the coach, and collective decisions were made regarding subsequent tactical modeling and game-direction adjustments.

### 2.12 Statistics

Data were analyzed using IBM SPSS Statistics (version 29.0; IBM Corp., Armonk, NY, USA). Descriptive statistics are presented as mean ± standard deviation (SD) and 95% confidence intervals (95% CI). The distribution of each variable was checked using the Shapiro–Wilk test, and the homogeneity of variances between groups was examined using Levene’s test. To examine the differences among the seven elements, a repeated-measures analysis of variance (ANOVA) was applied across microcycles. An independent samples t-test was used to compare the retraining and competition microcycles. A one-way repeated-measures ANOVA was performed to examine the differences in sRPE values across the five training days of the retraining and competition microcycles. Pairwise *post-hoc* comparisons were performed using paired sample t tests with Bonferroni adjustments. The magnitude of between-group differences was quantified using Cohen’s *d* effect size, interpreted as: trivial (< 0.20), small (0.20–0.49), moderate (0.50–0.79), or large (≥ 0.80). Associations between variables were assessed using Pearson’s correlation coefficient (r) and the corresponding *p*-values were reported. Lagged correlations were calculated between the sRPE (day t) and next day (t + 1) subjective mental state variable (fatigue, sleep quality, muscle soreness, stress, mood, and well-being) responses. Linear regression analysis was used to evaluate the predictive capacity of sRPE for subsequent neuromuscular performance.

To assess the competitive performance in a handball match, two performance outcomes were created: goal difference (the difference between goals scored and goals conceded) and match outcome (win = 1, loss = 0). For the binary match-outcome variable, point-biserial correlations were applied. This coefficient is mathematically equivalent to Pearson’s r when one variable is dichotomous. Statistical significance was set at *p* < 0.05.

## 3 Results

### 3.1 Results by training period

#### 3.1.1 External and internal loads led to the practical abandonment of traditional periodization

A total of 39 microcycles were analyzed, including 17 retraining and 22 competition microcycles (Tables 3, 4). Overall, compared to retraining microcycles, competition microcycles were characterized by longer duration and higher intra-microcycle variability, whereas the average sRPE, monotony, TWL, and strain were not significantly different. Pearson’s correlation analysis revealed several large positive associations among the principal weekly load indicators across the season. The minutes showed strong positive correlations with monotony (*r* = 0.883, *p* < 0.001), TWL (*r* = 0.879, *p* < 0.001), and strain (*r* = 0.895, *p* < 0.001). Similarly, monotony was strongly associated with the TWL (*r* = 0.863, *p* < 0.001) and almost perfectly correlated with strain (*r* = 0.983, *p* < 0.001). In addition, the TWL was strongly correlated with strain (*r* = 0.920, *p* < 0.001). However, the average sRPE showed a moderately positive correlation with TWL (*r* = 0.401, *p* = 0.011), indicating that a higher average perceived exertion was associated with a greater weekly training load. Thus, the seasonal variations of training duration in minutes, monotony, TWL, and strain were strongly interconnected, whereas the average sRPE demonstrated a more limited pattern of association, being significantly related only to TWL in the present dataset.

**Table 3:** Structure of periodization and variation of external and internal loads in microcycles throughout the season.

			Loads				
#	Blocks	Types	Minutes	Average sRPE	Monotony	TWL	Strain
1	1. Super Cup	Retraining	450	644.4 ± 121.8	1.45	3484	5055
2		Retraining	450	683.5 ± 72.6	1.44	3417	4925
3		Competition	445	657.4 ± 140.9	1.38	3287	4529
4	2. European Cup & LCh regular season - Part I	Retraining	450	649.2 ± 99.6	1.42	3246	4602
5		Competition	510	565.2 ± 111.3	2.05	3391	6944
6		Retraining	475	769.6 ± 98.4	1.43	3848	5508
7		Retraining	455	711.6 ± 50.5	1.45	3558	5172
8		Competition	460	599.8 ± 194.1	1.29	2999	3861
9		Competition	430	614.4 ± 213.9	1.26	3072	3885
10		Competition	420	608.2 ± 169.1	1.33	3041	4036
11		Competition	350	617.3 ± 164.7	1.01	2469	2489
12		Competition	470	651.6 ± 179.1	1.33	3258	4333
13		Competition	470	607.0 ± 75.2	1.43	3035	4350
14		Retraining	460	701.0 ± 102.7	1.42	3507	4986
15		Competition	465	615.8 ± 140.9	1.37	3079	4209
16		Retraining	450	630.8 ± 12.6	1.39	3154	4379
17		Competition	465	654.6 ± 86.9	1.43	3273	4677
18		Retraining	280	754.7 ± 127.1	0.79	2264	1786
19	3. LCh regular season - Part II & Lithuanian Cup	Rest					
20		Retraining	450	756.6 ± 118.8	1.42	3783	5356
21		Competition	555	686.2 ± 199.1	2.06	4117	8500
22		Retraining	450	710.0 ± 65.0	1.45	3550	5137
23		Retraining	450	694.0 ± 155.0	1.37	3472	4761
24		Competition	470	643.0 ± 58.0	1.45	3215	4654
25		Competition	555	645.0 ± 84.0	2.16	3870	8372
26		Retraining	360	545.0 ± 5.8	1.07	2160	2309
27		Competition	575	701.0 ± 146.6	2.02	4206	8514
28		Retraining	300	512.3 ± 166.8	0.98	2049	2012
29		Competition	550	541.7 ± 92.6	2.16	4125	8896
30		Competition	555	632.0 ± 80.2	2.17	3792	8222
31		Competition	430	632.8 ± 102.2	1.41	3164	4471
32		Competition	450	636.8 ± 99.7	1.42	3184	4509
33	4. LCh Final stage - Playoffs	Retraining	455	523.8 ± 293.0	1.45	3384	4916
34		Retraining	450	683.6 ± 76.2	1.44	3418	4919
35		Retraining	270	706.3 ± 45.1	0.80	2119	1695
36		Competition	540	609.5 ± 123.4	2.04	3657	7450
37		Retraining	330	519.3 ± 125.8	1.02	2077	2115
38		Competition	447	457.4 ± 336.2	1.10	2984	3286
39		Competition	483	606.5 ± 291.9	0.90	2426	2188
40		Competition	310	546.3 ± 330.1	0.67	1639	1101

LCh, Lithuanian championship; TWL, total weekly load.

**Table 4:** Indicators of external and internal loads in retraining and competition microcycles.

	Retraining microcycles		Competition microcycles			
Variable	Mean ± SD	95% CI	Mean ± SD	95% CI	*p*	Cohen’s *d*
Minutes	410.9 ± 71.2	374.3–447.5	473.0 ± 66.5	443.5–502.5	0.008	0.91
Average sRPE (AU)	658.6 ± 85.2	614.8–702.4	615.0 ± 52.5	591.7–638.2	0.056	0.64
Monotony (AU)	1.28 ± 0.24	1.16–1.41	1.52 ± 0.44	1.32–1.72	0.054	0.64
TWL (AU)	3087.6 ± 656.2	2750–3425	3240.1 ± 596.7	2975.6–3504.7	0.453	0.24
Strain (AU)	4096.1 ± 1434.2	3358.7–4833.5	5158 ± 4147.7	4147.7–6168.3	0.102	0.54

CI, confidence interval; SD, standard deviation; sRPE, session rating of perceived exertion; TWL, total weekly load.

**Figure 1:**
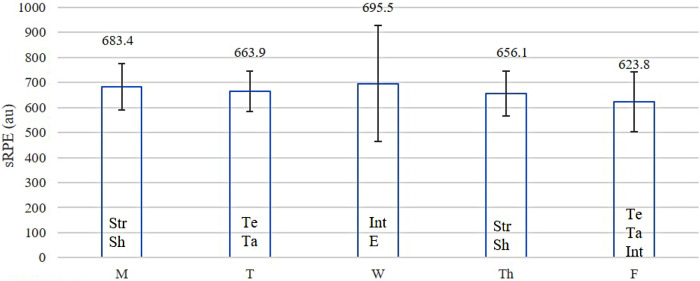
Training days. Retraining microcycle model: sRPE and training content. Daily sRPE presented in arbitrary units (au) in retraining microcycles (*n* = 17). Content: Str, strength; AeE, aerobic endurance; Te, technical; Ta, tactical; Int, integral; Sh, shooting.

Descriptive statistics and a comparative analysis of training content (expressed as a percentage of the total microcycle volume) were conducted for both the retraining and competition microcycles (Table 5). Repeated-measures ANOVA revealed a significant overall effect among the seven content elements across seasons (F(6, 228) = 22.34, *p* < 0.001), indicating a non-uniform distribution of training content between elements. Compared to retraining microcycles, competition microcycles contained significantly greater proportions of warm-up, integral, and theoretical content, whereas retraining microcycles showed significantly greater proportions of physical, technical, and recovery content. No significant differences were found in the tactical content. The daily internal training load (sRPE) during the retraining microcycle is shown in Figure 1.

**Table 5:** Differences in training content between retraining and competition microcycles (%).

	Retraining		Competition			
Content element	Mean ± SD	95% CI	Mean ± SD	95% CI	*p*	Cohen’s *d*
Warm-up	21.57 ± 2.91	20.08–23.07	24.75 ± 2.87	23.48–26.02	0.0017	1.10
Recovery	13.88 ± 3.22	12.23–15.53	11.27 ± 1.95	10.40–12.13	0.0068	−1.01
Integral	11.81 ± 8.74	7.31–16.30	20.18 ± 7.84	16.70–23.66	0.0040	1.02
Physical	18.54 ± 7.04	14.92–22.16	8.98 ± 7.38	5.71–12.25	0.0002	−1.32
Technical	22.49 ± 9.65	17.53–27.45	13.42 ± 7.14	10.26–16.59	0.0030	−1.09
Tactical	10.81 ± 9.06	6.15–15.47	15.53 ± 6.04	12.85–18.21	0.0752	0.63
Theoretical	0.89 ± 1.88	0.07–1.86	5.86 ± 8.69	2.01–9.72	0.0157	0.75

CI, confidence interval; SD, standard deviation.

The structure of the training content within the microcycle was organized such that strength and shooting were emphasized on Mondays and Thursdays, technical and tactical components on Tuesdays integrated training predominated on Wednesdays, and endurance and technical–tactical work, including game simulation against upcoming opponents, was conducted on Fridays. A one-way repeated-measures ANOVA indicated no significant differences in sRPE between weekdays (F(4, 48) = 0.94, *p* = 0.448, *η*p² = 0.073), suggesting a homogeneous distribution of internal load across the week. *post-hoc* comparisons demonstrated that mid-week sessions elicited significantly higher sRPE values than end-of-week sessions (*p* < 0.05), while differences between adjacent days were smaller and not consistently significantly different. The magnitude of differences between the highest and lowest load days corresponded to a moderate effect size (Cohen’s *d* = 0.5–0.8), suggesting practically meaningful variation in internal load distribution.

**Figure 2:**
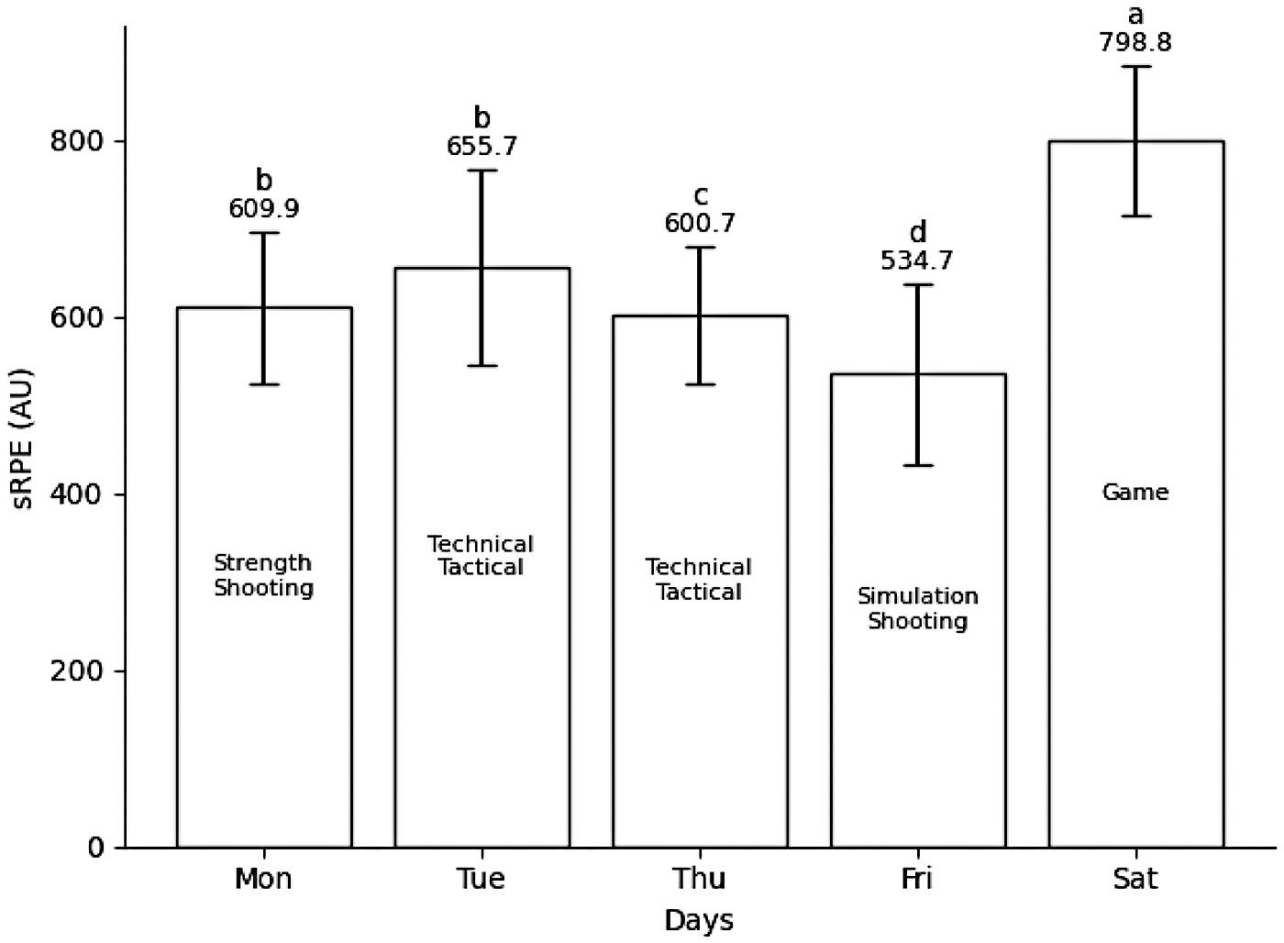
Internal load (sRPE) during the days of the competition microcycle (game on Saturday). Data are shown as mean ± SD (*n* = 17). The letters above the bars indicate statistically significant differences between days (*p* < 0.05). sRPE, session rating of perceived exertion.

sRPE differed across competition microcycle days when matches were played on Saturdays (Figure 2).

The microcycle consisted of four training sessions and one match. Strength development and shooting were prioritized on Mondays, whereas technical and tactical skills were emphasized on Tuesdays and Thursdays. The Friday session was dedicated to simulating upcoming match play scenarios and shooting practice. A significant main effect of day on the sRPE was observed (*F* = 20.40, *p* = 0.000). The highest load was observed on game day (Saturday), whereas the lowest values were recorded during the technical–tactical (Thursday) and simulation sessions (Friday). Bonferroni-adjusted paired comparisons showed that Saturdays were significantly higher than Mondays (*p* = 0.000, d_z = 1.39), Tuesdays (*p* = 0.032, d_z = 0.84), Thursdays (*p* < 0.001, d_z = 1.59), and Fridays (*p* < 0.001, d_z = 2.02). In addition, Tuesdays were significantly higher than Fridays (*p* = 0.001, d_z = 1.29). No other pairwise differences were statistically significant after Bonferroni correction.

**Figure 3:**
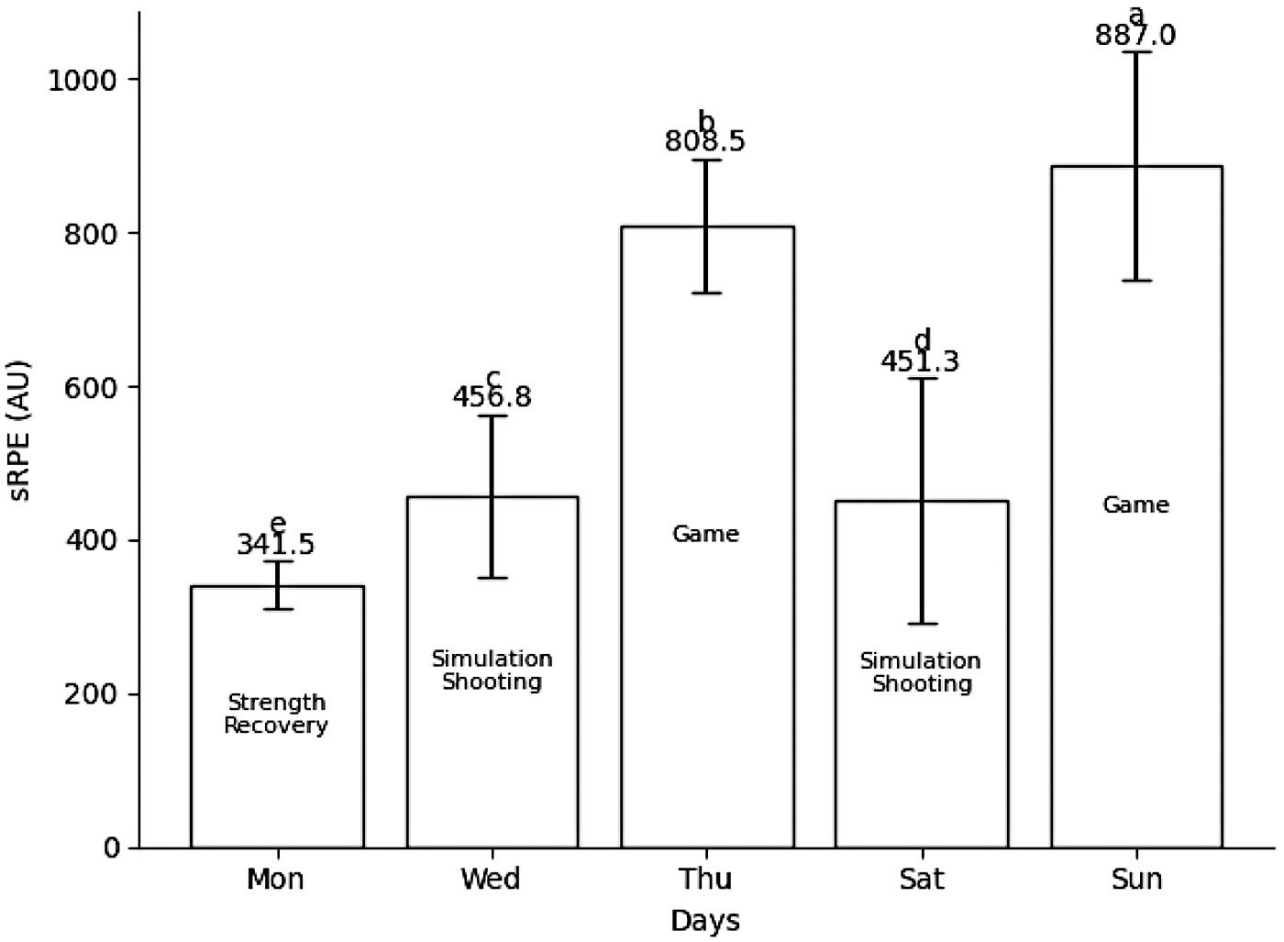
Internal load (sRPE) during the days of the competition microcycle (matches on Thursday and Sunday). Data are shown as mean ± SD (*n* = 5). sRPE, session rating of perceived exertion.

During the final stage, when competing for rankings, two matches were scheduled per week (Thursday and Sunday). Accordingly, match play was simulated on the preceding days (Wednesday and Saturday), whereas Tuesday was allocated as the rest day. A significant effect of day on the sRPE was observed (*F* = 17.61, *p* = 0.000). Mean sRPE ranged from 341.5 to 887.0 AU; Figure 3). Game days showed the highest internal load, whereas recovery-oriented sessions showed the lowest values.

### 3.2 Throw velocity, body composition, and physical performance adaptations across the season

Changes between the first and last measurement were evaluated using absolute differences (Δ), percent changes (%), and effect sizes (Cohen’s *d*). Effect sizes were interpreted according to Hopkins’ scale (trivial < 0.2; small 0.2–0.6; moderate 0.6–1.2; large 1.2–2.0; or very large > 2.0). The throwing velocity increased across the season (Table 6). Effect sizes ranged from moderate to large, indicating significant changes. Substantial improvements were observed in all core and upper body endurance variables, and in YYIE1 performance and the VO_2_max across the season. The changes in the CMJ, body mass, and fat mass were small and not statistically significant.

**Table 6:** Descriptive statistics of throwing velocity, endurance and strength variables, and CMJ.

Variable	Pre	Post	*Δ*	%	t	*p*	Cohen *d*	Hopkins
Three-step shot (km^.^ h^–1^)	72.9 ± 2.1	77.1 ± 2.2	4.20	5.76	5.07	<0.001	1.95	Large
Jump shot (km^.^ h^–1^)	74.4 ± 2.2	78.9 ± 2.5	4.50	6.05	5.06	<0.001	1.91	Large
Standing shot (km^.^ h^–1^)	70.1 ± 2.1	72.6 ± 2.1	2.50	3.57	3.15	<0.001	1.19	Moderate
Body mass (kg)	70.7 ± 7.2	67.9 ± 6.3	−2.80	−3.96	–1.1	*p* = 0.284	−0.41	Small
Fat (%)	18.5 ± 2.1	17.5 ± 1.7	−1.00	−5.41	–1.38	*p* = 0.178	−0.52	Small
YYIE1 (m)	1375 ± 754	2747 ± 225	1372	99.78	6.52	<0.001	2.47	Very large
VO_2_max (ml^.^ kg–^1.^ min–^1^)	43.3 ± 3.7	48.6 ± 5.8	5.30	12.24	2.88	*p* = 0.008	1.09	Moderate
Plank hold (s)	69.0 ± 19.2	151.0 ± 71.8	82.00	118.84	4.13	<0.001	1.56	Large
Flexed arm hang (s)	19.2 ± 13.3	32.2 ± 12.1	13.00	67.71	2.71	*p* = 0.012	1.02	Moderate
CMJ (cm)	35.1 ± 4.7	37.3 ± 6.5	2.20	6.27	1.03	*p* = 0.314	0.39	Small

CMJ, countermovement jump; VO_2max_, maximal oxygen uptake; YYIE1, Yo-Yo intermittent endurance test level 1.

### 3.3 Associations among previous-day sRPE, next-day CMJ, and subjective well-being

The previous day’s training load, quantified using the sRPE metric, was considered the load of the preceding session and examined in relation to the next day’s CMJ performance and subjective mental state variables. No significant lagged relationships were observed between the sRPE and next-day CMJ or well-being variables. Pearson correlations ranged from *r* = −0.062 to *r* = 0.088 (*p* > 0.05). Specifically, no significant correlations were found between sRPE_t and CMJ_t + 1 (*r* = −0.062, *p* = 0.392), fatigue (*r* = 0.088, *p* = 0.225), sleep quality (*r* = −0.077, *p* = 0.289), muscle soreness (*r* = 0.022, *p* = 0.758), stress (*r* = 0.001, *p* = 0.989), mood (*r* = −0.054, *p* = 0.460), or overall well-being (*r* = −0.000, *p* = 0.999). Linear regression analysis confirmed the absence of a predictive relationship between training load and neuromuscular performance on the next day. The regression analysis confirmed no predictive effect (*R*² = 0.004, *p* = 0.392). The model was not statistically significant (*F* = 0.737, *p* = 0.392), with sRPE_t not predicting CMJ_t + 1 (*β* = −6.79, *p* = 0.392). The explained variance was negligible (*R*² = 0.004), indicating that the training load had no meaningful predictive value for the next day’s neuromuscular status. The retraining microcycles were characterized by a slightly higher training load (sRPE) than the competition microcycles (*p* = 0.041, ES = 0.22), whereas no differences were observed in CMJ performance (*p* = 0.62). However, well-being scores were significantly lower during the competition microcycles (*p* = 0.048, ES = 0.22). Correlation analysis revealed no significant associations between sRPE and the next-day CMJ or well-being during the retraining microcycles. In contrast, a small but significant negative association between sRPE and well-being was observed during competition microcycles (*r* = –0.21, *p* < 0.05).

### 3.4 Seasonal changes in monotony, TWL, strain, and A:CWR

**Figure 4:**
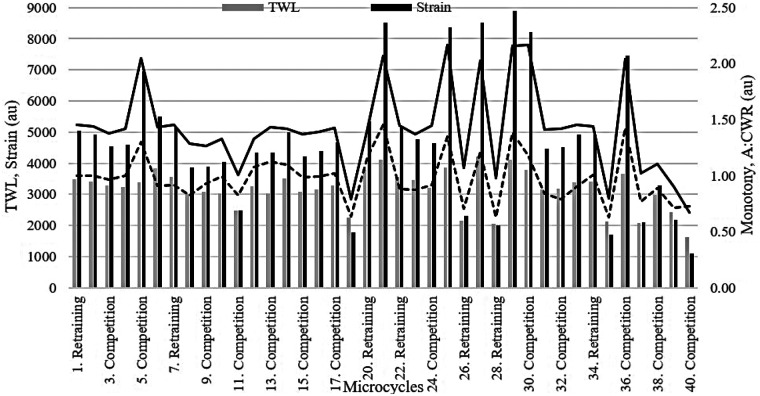
Temporal changes in monotony, total weekly load (TWL), strain, and acute:chronic workload ratio (A:CWR) throughout the season.

The indicators recorded over 40 microcycles allowed to determine normative values of monotony (1.42 ± 0.39; 95% CI, 1.29–1.54), TWL (3173 ± 619; 95% CI, 2972–3374), strain, (4695 ± 2005; 95% CI, 4045–5345), and A:CWR (0.974 ± 0.035; 95% CI, 0.905–1.044). Monotony ranged from a low of 0.671 AU during the 40th competition microcycle and from 0.789 AU during the 18th retraining microcycle to a max of 2.163 AU during the 25th competition microcycle and also exceeded the 2 AU value in the 5th, 21st, 27th, 29th, 30th, and 36th competition microcycles. The TWL ranged from low 1639 AU during the final 40th competition microcycle and 2119 AU during the 35th retraining microcycle to a max of 4206 AU during the 27th competition microcycle. Strain ranged from a low of 1101 AU during the 40th competition microcycle and 1695 AU during the 35th retraining microcycle to a max of 8896 AU during the 29th competition microcycle and also exceeded the 8000 AU values in the 21st, 27th, and 30th competition microcycles. The A:CWR ranged from a low of 0.626 during the 35th retraining microcycle, to a max 1.451 AU during the 21th competition microcycle (Figure 4). Pearson’s correlation analysis revealed a strong relationships between monotony and strain (*r* = 0.98, *p* < 0.001) and between TWL and strain (*r* = 0.92, *p* < 0.001), confirming that strain integrates both load magnitude and variability. In contrast, the A:CWR-derived variables demonstrated weaker and inconsistent associations, with several correlations not reaching statistical significance (*p* > 0.05).

### 3.5 Match performance, training content, and match outcome

Only matches with a final goal difference of fewer than seven were included in the analytical dataset (*n* = 13). Across the 13 matches, the team won ten and lost three. Table 7 presents the rankings of the top correlation indicators and descriptive statistics for all absolute and percentage-based indicators. To assess outcome-related relationships, the outcome of the match was coded as win = 1 and loss = 0. For the binary match-outcome variable, point-biserial correlations were applied. This coefficient is mathematically equivalent to Pearson’s r when one variable is dichotomous. The strongest positive correlations were identified for efficiency of positional defense in the first half (*r*ₚᵦ = 0.680, *p* = 0.011) and overall defensive efficiency in the first half (*r*ₚᵦ = 0.653, *p* = 0.016). Additionally, goalkeeper efficiency (total) was significantly associated with match outcome (*r*ₚᵦ = 0.576, *p* = 0.039), and goalkeeper efficiency in the first half (*r*ₚᵦ = 0.533, *p* = 0.061). Similarly, overall defensive efficiency across matches showed a moderate relationship (*r*ₚᵦ = 0.523, *p* = 0.067). Offensive and transitional indicators demonstrated weaker associations with the matched outcomes. Variables such as steals (*r*ₚᵦ = 0.452), first-half goals (*r*ₚᵦ = 0.432), and fast break efficiency in the first half (*r*ₚᵦ = 0.428) showed moderate but non-significant relationships (*p* > 0.05). Overall, the ranked correlation structure indicated that defensive efficiency variables, particularly those in the first half, exhibited the strongest associations with match outcomes, whereas offensive indicators showed comparatively smaller and less consistent relationships.

**Table 7:** Descriptive statistics (mean ± SD, 95% CI) per match and point-biserial correlations between performance indicators and match outcome (win/loss).

Variable	r_pb	*p*	Mean	SD	95% CI (Lower)	95% CI (Upper)
Positional defensive efficiency 1 halftime, %	0.680	0.011	57.34	11.89	50.16	64.53
Defensive efficiency 1 halftime total, %	0.653	0.016	55.32	9.33	49.68	60.96
Goalkeepers’ efficiency total, %	0.576	0.039	36.98	6.14	33.26	40.69
Goalkeepers’ efficiency 1 halftime, %	0.533	0.061	33.91	9.33	28.27	39.54
Defensive efficiency total, %	0.523	0.067	56.31	6.39	52.44	60.17
Positional defensive efficiency total, %	0.497	0.084	58.89	7.65	54.27	63.51
Goalkeepers’ efficiency wings shots, %	0.468	0.107	50.77	29.97	32.66	68.88
Steals	0.452	0.121	4.00	1.68	2.98	5.02
Goals 1 halftime	0.432	0.140	15.85	3.31	13.84	17.85
Goals fastbreaks 1 halftime	0.429	0.143	6.31	3.07	4.46	8.16
Efficiency fastbreaks 1 halftime, %	0.428	0.145	54.47	12.12	47.15	61.79

CI, confidence interval; r_pb, point-biserial correlation coefficient; SD, standard deviation.

The training content variables (physical, technical, tactical, integral, theoretical, warm-up, and recovery) were treated as continuous variables. The relationships between the training content and match outcomes were assessed using point-biserial correlation coefficients (r_pb), which are equivalent to Pearson’s correlations when one variable is dichotomous. Differences between matches that were won or lost were evaluated using independent samples t-tests. The match outcome was primarily associated with acute training content (i.e., the last microcycle prior to competition), whereas chronic training content (seasonal averages) demonstrated weaker and non-significant relationships (*p* > 0.05). Point-biserial correlation analysis revealed that tactical training exhibited the strongest positive relationship with match outcomes (*r* = 0.54, *p* = 0.055), followed by integral training (*r* = 0.48, *p* = 0.095). Moderately positive relationships were observed for technical (*r* = 0.31, *p* = 0.34) and physical training at the chronic level (*r* = 0.28, *p* = 0.37), although these relationships were not statistically significant. Integral training demonstrated a moderate-to-large difference between the groups (*p* = 0.082), indicating a meaningful trend. Tactical training demonstrated a large effect size between won and lost matches (ES = 0.95), whereas integral training showed a moderate to large effect (ES = 0.82).

## 4 Discussion

The present study aimed to investigate the temporal dynamics and interrelationships among key training and performance variables across a competitive season and to establish reference ranges for integrated monitoring variables to support adaptive, data-informed microcycle adjustments in semi-professional female handball. The main findings indicate that integrated monitoring provides a sensitive framework for capturing fluctuations in training load, athlete responses, and performance throughout the season.

### 4.1 Seasonal dynamics of training load and microcycle structure

The competition schedule and its hierarchical structure during the season led to the practical abandonment of traditional periodization, not only traditional classical linear periodization but also a block periodization model. The competition schedule and its hierarchical structure during the season necessitated abandoning traditional classical linear periodization as well as a block periodization model. Instead, the season was organized into four competition blocks of unequal duration. Within each block the alteration between the retraining and competition microcycles was adjusted according to the competitive calendar (Table 3). The shortest block (three microcycles) occurred at the beginning of the season and was associated with participation in the Super Cup, when the competition occurred after only two retraining microcycles. The second block, which included participation in the European Cup and the first round of the Lithuanian Championship Regular Season, comprised 14 microcycles: five retraining and nine competition. The third block consisted of 15 microcycles, including eight retraining, six competition, and one rest. Finally, during the Lithuanian Championship playoffs held at the end of the season, the fourth block consisted of eight microcycles unevenly distributed between four retraining and four competition.

A central finding of the present study is that the seasonal training process was primarily shaped by competition demands rather than by a predefined periodization model, as evidenced by the systematic differences between retraining and competition microcycles. Competition microcycles were characterized by significantly higher total training volume (473.0 ± 66.5 vs. 410.9 ± 71.2 min; *p* = 0.008; ES = 0.91), accompanied by a tendency toward lower average internal load (615.0 ± 52.5 vs. 658.6 ± 85.2 AU; *p* = 0.056; ES = 0.64) and higher monotony (1.52 ± 0.44 vs. 1.28 ± 0.24; *p* = 0.054; ES = 0.64). This pattern indicates that the competition phases were dominated by greater volume but reduced stimulus variability, reflecting externally imposed match constraints rather than structured load progression.

From a mechanistic perspective, these findings highlight the importance of training load dynamics, rather than load magnitude alone. According to Foster et al.’s model (20), monotony reflects the day-to-day variability of the load, whereas strain represents the combined effect of load magnitude and monotony (22). In the present study, the increase in monotony and tendency toward higher strain during competition microcycles (5158 vs. 4096 AU; ES = 0.54) indicated reduced variability and increased cumulative physiological stress. Notably, several competition microcycles exhibited monotony values exceeding 2.0 and strain values above 8500 AU, suggesting periods of heightened fatigue risk. These results demonstrate that competition density constrains variability, which is a key principle in effective periodization.

Importantly, while the TWL did not differ significantly between microcycle types (*p* = 0.453), its internal structure changed substantially. This indicates that handball performance is not driven by increasing the load magnitude, but by how the load is distributed and organized across the microcycle. In contrast to the competition phases, the retraining microcycles were characterized by greater variability in daily load, which was reflected in lower monotony values, suggesting a more flexible and adaptive training structure. This supports the contemporary view that training processes should be dynamically adjusted rather than rigidly preplanned (9, 10, 59, 60).

Analysis of the training content further reinforced this competition-driven restructuring. During competition microcycles, there was a significant shift toward integral and game-based activities (20.18% vs. 11.81%; *p* = 0.004; ES = 1.02) and warm-up (24.75% vs. 21.57%; *p* = 0.0017; ES = 1.10), while physical (8.98% vs. 18.54%; *p* = 0.0002; ES = −1.32) and technical training (13.42% vs. 22.49%; *p* = 0.003; ES = −1.09) were markedly reduced. This demonstrates a clear shift from performance development to performance regulation, where the goal is to maintain readiness rather than to induce new adaptations. These findings also help explain the limitations of the traditional and block periodization models in handball. While such models assume that training stimuli can be systematically sequenced to produce predictable adaptations (61), the present data show that training is continuously reorganized in response to match demands. This finding supports previous research demonstrating that different periodization models can produce similar outcomes in handball (11), suggesting that adaptation depends more on contextual alignment than on the model.

A critical implication of the present study is the emergence of a load–response paradox during the season. On one hand, the increased monotony and strain during competition microcycles indicate elevated fatigue risk; on the other hand, the reductions in physical and technical training suggest limited stimuli for adaptation. This creates a narrow “optimal load window,” in which both excessive and insufficient load can negatively impact performance. In this context, maintaining performance requires continuously balancing the stimuli and recovery rather than reliance on discrete training phases. The observed training structure reflects principles consistent with microcycle periodization and microdosing, in which the load is distributed throughout the week in smaller targeted stimuli (17, 18). The moderate effect sizes for the sRPE and monotony suggest that the load was regulated within a relatively narrow range, which was likely to prevent excessive fatigue accumulation while maintaining performance.

Finally, the behavior of the A:CWR during the season observed in the present study should be interpreted with caution. Although originally proposed as a predictor of injury risk, recent studies have identified substantial methodological and conceptual limitations (24). Therefore, in the context of the present findings, the A:CWR should be considered a descriptive indicator of load dynamics rather than a predictive tool, particularly given the dominance of competition-driven load fluctuations. This interpretation aligns with the current scientific consensus and reinforces the importance of using multiple monitoring indicators (14).

Taken together, the present findings demonstrate that periodization in handball cannot be effectively implemented as a rigid, preplanned structure. Instead, it has emerged as a dynamic, competition-driven process in which the training load, variability, and content are continuously adjusted in response to match demands. In this framework, the primary objective shifts from maximizing adaptation through structured phases to maintaining performance through optimal regulation of training stress across the competition season.

### 4.2 Integrated athlete responses and performance adaptations: neuromuscular, perceptual, strength, endurance, and throwing velocity

The present findings indicate that athletes’ responses to training load should be interpreted across at least two timescales: short-term regulation of readiness and long-term accumulation of sport-specific adaptation. In the present sample, CMJ changed slightly and non-significantly across the season (+2.20 cm, +6.27%, *p* = 0.314, *d* = 0.39), whereas competition microcycles were associated with lower well-being (*p* = 0.048; ES = 0.22), and sRPE showed a small negative association with well-being during competition microcycles (*r* = −0.21, *p* < 0.05). Simultaneously, no meaningful relationships were observed between the previous-day sRPE and the next-day CMJ or well-being (*r* = −0.062–0.088; regression *R*² = 0.004). This pattern suggests that in these handball players, the CMJ was relatively stable across the season and, therefore, was more useful for contextualizing in-season readiness than for tracking chronic seasonal improvement. This interpretation is supported by previous research indicating that the CMJ is a valid and sensitive tool for monitoring neuromuscular status and fatigue but may not fully reflect long-term performance adaptations or sport-specific development (24–26, 40, 62).

Perceptual findings are also important and should be interpreted against existing monitoring literature. Saw et al. (25) concluded that subjective self-reported measures tend to reflect training stress with greater sensitivity and consistency than many commonly used objective markers, particularly when athletes are exposed to accumulating loads. Specifically, Henze et al. (63) showed that while objective and subjective recovery-related markers are associated, they are not interchangeable, indicating that they capture different dimensions of the athlete’s physical and mental state. Accordingly, the present divergence between a relatively unchanged CMJ and impaired well-being during competition microcycles should not be viewed as contradictory. Rather, the divergence suggests that the competition phase imposed a burden that was registered earlier or more clearly at the perceptual level than in the lower-limb jump output. In practical terms, these findings support the use of subjective well-being measures as sensitive daily indicators of accumulated training and competition stress, whereas CMJ, although a valid marker of neuromuscular status, should be interpreted cautiously and within context because of its limited ability to fully capture the multifactorial nature of athlete adaptation (26, 27, 64).

The absence of meaningful associations between sRPE and next-day CMJ or well-being deserves careful interpretation. These findings do not indicate that the training load is unimportant. Rather, they suggest that athlete responses are not governed by a simple single-session cause–effect mechanism. Instead, readiness appears to emerge from the combined influence of competition density, microcycle organization, cumulative fatigue, and recovery processes, which is consistent with integrated and multifactorial models of adaptation rather than linear load–response frameworks (10, 65).

In contrast to these short-term response patterns, this study demonstrated substantial chronic performance adaptations. Throwing velocity improved in all three conditions, with the largest effects in the three-step throw (+5.76%; *d* = 1.95) and jump shot (+6.05%; *d* = 1.91) conditions, whereas standing throw velocity increased (+3.57%; *d* = 1.19). These changes are consistent with the handball literature indicating that throwing velocity is a trainable sports-specific quality and that higher-level players typically demonstrate superior throwing performance. Wagner et al. (66) reported that elite handball players show superior biomechanical and physiological performance profiles compared to lower-level players, while van den Tillaar (67) concluded that overarm throwing velocity responds to both strength- and velocity-oriented interventions. Therefore, the increase in throw velocity is most plausibly explained by the combined effect of repeated sport-specific throwing exposure, improved segmental coordination, and better force transfer under match-like conditions rather than by a single isolated fitness factor. The simultaneous improvement in throwing velocity and strength endurance variables provides a plausible mechanistic basis for this interpretation. In the present study, plank-hold increased by 118.84% (*d* = 1.56) and flexed-arm hanging increased by 67.71% (*d* = 1.02). Although the present study did not test direct within-sample correlations between throwing velocity and strength measures, previous studies have demonstrated that upper body and shoulder strengths are significantly associated with ball velocity in handball players, whereas trunk-focused training interventions can further enhance throwing performance by improving force transmission through the kinetic chain (68, 69).

Endurance-related adaptations are also substantial and highly relevant to handball performance. YYIE1 improved by 99.78% (*d* = 2.47), and VO₂max increased by 12.24% (*d* = 1.09). Michalsik et al. (70) showed that elite female handball players experience repeated high-intensity locomotor and technical demands supported by a considerable aerobic contribution, making intermittent endurance and aerobic recovery capacity central to sustaining match performance. Against this background, the present findings suggest that even in competition-dominated seasons, appropriately distributed training and repeated match exposure can induce meaningful endurance adaptations. This is important because it indicates that handball players do not necessarily require a long classical preparatory phase to improve their intermittent and aerobic capacities provided that the seasonal load structure is sufficiently coherent. This interpretation is supported by evidence demonstrating that in season and concurrent training can maintain or improve physiological capacities in handball players (42); adaptation can be effectively driven through microcycle-based load organization; and performance development depends on integrated, multifactorial training processes rather than strictly predefined preparatory phases (65). Furthermore, the high physiological demands of handball match play may contribute to these adaptations (69).

A key implication of the present data is that for these players, performance development throughout the season was not primarily expressed through improvements in explosive lower-limb jump output. The lack of parallel improvement in the CMJ, despite clear gains in throwing velocity, endurance, and strength endurance, suggests that the dominant adaptations in this sample were likely sport-specific, metabolic, and upper body/trunk-related. This interpretation is supported by previous research indicating that CMJ is a sensitive marker of neuromuscular status and fatigue but does not necessarily reflect all dimensions of performance development (26–28). In contrast, handball-specific performance, particularly throwing velocity, has been shown to depend on a multidimensional interaction of technical, coordinative, and strength-related factors rather than on a single general power indicator. Therefore, in the present study, CMJ should not be overinterpreted as a universal marker of progress throughout the season but rather considered as a component within a broader, integrated monitoring framework.

Taken together, the present findings support an integrated interpretation in which CMJ and well-being inform short-term fatigue management, whereas throwing velocity, YYIE1, VO₂max, and strength endurance reflect long-term adaptation. This distinction helps explain why acute monitoring variables did not directly predict performance improvements over the season and reinforces the practical need to combine day-to-day monitoring with periodic sport-specific testing. Such an integrated approach is fully aligned with current multifactorial periodization frameworks, which argue that effective performance management in team sports should incorporate training load, recovery, fitness, and context rather than rely on any single marker (14).

### 4.3 Linking monitoring variables to match performance

From an outcome perspective, the present findings indicate that match success in this sample was driven primarily by defensive performance variables rather than offensive volume indicators. The strongest point-biserial associations with winning were observed for efficiency in positional defense in the first half (r_pb = 0.680, *p* = 0.011), overall first half defensive efficiency (r_pb = 0.653, *p* = 0.016), and total goalkeeper efficiency (r_pb = 0.576, *p* = 0.039). The won versus lost comparison was consistent with these correlations. Won matches were characterized by fewer goals conceded, higher total defensive efficiency, and better first half defensive control. In other words, the probability of winning for this team depended more on limiting the opponent’s scoring opportunities and stabilizing the match earlier than on simply producing a high attack volume. This interpretation is important because much of the handball match analysis literature has historically concentrated on offensive actions, total shots, final scores, and broad outcome descriptors, while explicitly leaving defensive performance comparatively underdeveloped as an analytical field (2). A broad review of team ball sports reached a similar conclusion in which match analysis expanded considerably, but the dominant focus remained on technical and tactical event descriptions, with limited integration of wider performance models and an evident need for more holistic approaches (2). Against this background, the present study contributes original evidence showing that, in this semi-professional female player sample, defensive efficiency was not merely a secondary descriptor of performance but a central outcome-related mechanism.

To interpret this performance structure further, a comparison with elite female handball player benchmarks was conducted (Table 8). The elite comparison analysis clarified this performance model. Compared with elite team benchmarks, Žalgiris displayed a substantially higher number of attacks and a much greater fast break frequency, but lower overall attack efficiency, lower positional attack efficiency, and lower fast break efficiency (Table 8). This means that Žalgiris was not characterized by the efficiency-dominant profile typically seen in top-level teams. Rather, the team combined high-tempo, transition-oriented play with a more uneven efficiency structure. This contrast is compatible with previous elite handball team analyses showing that winning teams are generally distinguished by their superior shot effectiveness, more effective positional attacks, and stronger goalkeeper contributions (30–32, 71). In the 2022 Women’s European Championship, for example, winning teams showed higher attack effectiveness and higher goalkeeper defense values, while logistic regression models identified attack effectiveness and goalkeeper defenses as significant contributors to win probability (30). Likewise, elite club analyses have shown that winning teams tend to outperform losing teams in terms of offensive effectiveness, especially in positional and fast attacks, assistance, and finishing efficiency (31, 71). However, the present study also suggests that Žalgiris should not be interpreted simply as an offensively weaker version of elite teams. The internal outcome analysis showed that the team’s own winning mechanism was distinctly defense-led. This is where a comparison with recent defense-specific evidence becomes especially valuable. González-García’s (72) study of elite handball teams demonstrated that defensive success is strongly associated with concrete game variables such as forced offensive fouls, ball recovery and steals, blocked shots, passive play situations, numerical superiority, and the temporal organization of the defensive phase. These findings support the interpretation that defense in handball is not a passive, reactive component of play but an active regulatory system that shapes what the opponent can achieve. In the present study, this logic is reflected in the clear importance of first half defensive efficiency. Early defensive control likely reduced opponent-attacking fluency, improved goalkeeper conditions, and increased the probability of entering the remainder of the match from a position of tactical stability.

**Table 8:** Žalgiris vs. elite female handball performance model.

Variable	Žalgiris Mean ± SD	Elite Mean ± SD	Difference	*p*	ES (d)	Magnitude	Interpretation
Number of attacks	67.19 ± 5.26	53.64 ± 5.13	+13.55	<0.001	+2.63	very large	Žalgiris was significantly higher than elite.
Number of goals	31.23 ± 3.49	29.43 ± 4.45	+1.80	0.139	+0.42	small	Žalgiris was non-significantly higher than elite.
Attacks efficiency total	47.19 ± 4.21	54.14 ± 5.69	−6.95	<0.001	−1.29	large	Žalgiris was significantly lower than elite.
Number positional attacks	45.31 ± 5.95	48.45 ± 3.85	−3.14	0.093	−0.71	moderate	Žalgiris was non-significantly lower than elite.
Goals in positional attacks	21.08 ± 3.48	25.20 ± 3.76	−4.12	0.001	−1.11	moderate	Žalgiris was significantly lower than elite.
Efficiency positional attacks	44.99 ± 5.81	51.95 ± 6.44	−6.96	0.001	−1.10	moderate	Žalgiris was significantly lower than elite.
Number fast breaks	21.88 ± 6.84	4.34 ± 2.68	+17.54	<0.001	+4.41	very large	Žalgiris was significantly higher than elite.
Fast breaks goals	10.15 ± 2.91	3.16 ± 2.37	+6.99	<0.001	+2.80	very large	Žalgiris was significantly higher than elite.
Fast breaks efficiency	51.88 ± 8.97	67.80 ± 29.05	−15.92	0.003	−0.61	moderate	Žalgiris was significantly lower than elite.
Goals	33.19 ± 5.26	29.43 ± 4.45	+3.76	0.033	+0.81	moderate	Žalgiris was significantly higher than elite.
Shots efficiency	60.89 ± 6.38	54.14 ± 5.69	+6.75	0.003	+1.15	moderate	Žalgiris was significantly higher than elite.
Goals positional	21.38 ± 4.29	23.34 ± 2.03	−1.96	0.135	−0.70	moderate	Žalgiris was non-significantly lower than elite.
Shots positional efficiency	58.53 ± 11.60	51.95 ± 6.44	+6.58	0.070	+0.84	moderate	Žalgiris was non-significantly higher than elite.
Goals fast break	11.19 ± 5.08	3.44 ± 1.55	+7.75	<0.001	+2.68	very large	Žalgiris was significantly higher than elite.
Shots fast break efficiency	71.22 ± 9.68	67.80 ± 29.05	+3.42	0.508	+0.13	trivial	Žalgiris was non-significantly higher than elite.
Goals 9m	4.14 ± 2.28	5.59 ± 1.43	−1.45	0.048	−0.86	moderate	Žalgiris was significantly lower than elite.
Shots 9 m efficiency	40.51 ± 19.96	46.91 ± 18.73	−6.40	0.316	−0.34	small	Žalgiris was non-significantly lower than elite.
Shots 6 m efficiency	60.07 ± 16.22	71.45 ± 19.47	−11.38	0.045	−0.61	moderate	Žalgiris was significantly lower than elite.
Shots 7 m efficiency	86.60 ± 14.24	84.71 ± 18.08	+1.89	0.697	+0.11	trivial	Žalgiris was non-significantly higher than elite.
Goals wings	5.38 ± 1.91	5.84 ± 1.89	−0.46	0.464	−0.24	small	Žalgiris was non-significantly lower than elite.
Shots wings efficiency	52.76 ± 30.98	59.92 ± 14.33	−7.16	0.433	−0.37	small	Žalgiris was non-significantly lower than elite.
Goals majority	2.88 ± 1.78	3.49 ± 0.88	−0.61	0.256	−0.52	small	Žalgiris was non-significantly lower than elite.
Goals minority	4.13 ± 2.17	1.90 ± 0.71	+2.23	0.003	+1.78	large	Žalgiris was significantly higher than elite.
Goalkeeper efficiency total	36.98 ± 6.14	33.70 ± 7.88	+3.28	0.127	+0.44	small	Žalgiris was non-significantly higher than elite.
Goalkeeper efficiency wings	48.19 ± 28.57	37.39 ± 18.50	+10.80	0.218	+0.51	small	Žalgiris was non-significantly higher than elite.
Goalkeeper efficiency 9m	47.14 ± 12.76	49.95 ± 21.92	−2.81	0.565	−0.14	trivial	Žalgiris was non-significantly lower than elite.
Goalkeeper efficiency 7m	22.76 ± 20.39	21.58 ± 24.34	+1.18	0.863	+0.05	trivial	Žalgiris was non-significantly higher than elite.
Goalkeeper efficiency 6m	31.32 ± 14.20	33.53 ± 22.30	−2.21	0.672	−0.11	trivial	Žalgiris was non-significantly lower than elite.

ES, effect size; SD, standard deviation.

From a theoretical perspective, these findings align well with recent conceptualizations of handball as a complex, dynamic system. Espoz-Lazo and Hinojosa-Torres (2) argued that modern handball should be understood through nonlinearity, self-organization, emergent behaviors, and interacting constraints rather than through isolated technical variables. Viewed through this lens, defense can be interpreted as a system of tactical control that manipulates spatial, temporal, and informational constraints. Spatially, an organized defense reduces exploitable gaps and channels attack behavior into less favorable zones. Temporally, it disrupts rhythm, delays completion, and increases the likelihood of passive play or low-quality late possession solutions. Informationally, it reduces the attacker’s perceptual clarity and decision time, thereby increasing the uncertainty and error probability. The present results fit this model closely. Defensive efficiency was associated not only with lower concessions but also with match control, especially in the first half, suggesting that defense functions as a stabilizing subsystem within the broader game ecology.

Performance model identified in the present study:

Defensive efficiency (particularly in the first half) → fewer goals conceded → improved tactical control → increased transition opportunities → higher probability of winning.

This also explains the apparent coupling between defense and transition. Žalgiris produced very high fast break frequencies relative to elite reference values, yet its fast break efficiency was lower than other elite teams. This profile suggests that the transition volume was probably generated, at least in part, by successful defensive moments but that the conversion quality of these opportunities remained below the elite level (30, 31). Therefore, the team’s practical development pathway was not to abandon its transition identity but to preserve the defensive base that generates these opportunities while improving the efficiency with which they are converted. In applied terms, the current evidence supports the model in which defense reduced the number of goals conceded, improved tactical control and increased transition opportunities resulting in a higher win probability. This model is fully compatible with ecological and systems-based interpretations of team performance, in which attack and defense are not independent compartments but reciprocally coupled processes (2, 72).

The training monitoring findings were consistent with this interpretation. Acute pre-match tactical content and integral training showed the clearest positive relationships with match outcomes, whereas chronic content distribution was less informative. Taken together with the match performance results, this suggests that immediate game-specific preparation may have helped organize the defensive collective, sharpen situational coordination, and improve the quality of responses under competitive constraints. In practical coaching terms, these data support prioritizing tactical and integral content that reinforces collective defensive behavior, goalkeeper–defense cooperation, first half readiness, and the quality of transition decisions rather than focusing only on overall training volume.

Overall, this study advances the discussion in two ways. First, for this team, winning was more strongly linked to defense and goalkeeper performance than to offensive quantity. Second, it demonstrates that Žalgiris differed from other elite teams based not only on isolated indicators but also by exhibiting a different performance architecture. The team had an energetically high-volume, transition-rich model whose competitive success depended on defensive stability. Given that the literature on handball contains relatively few studies in which defense itself is treated as the primary explanatory mechanism of the outcome, this represents one of the main conceptual strengths of the present study (2, 71, 72).

### 4.4 Practical applications

The present study provides an applied framework for integrating training and monitoring processes throughout a handball competitive season.

First, coaches may use the reported reference values of internal load (session duration × sRPE), total weekly load (TWL), monotony, strain, and A:CWR as contextual benchmarks when planning and evaluating training programs. However, these values should be interpreted relative to the specific characteristics of the athlete group and competitive environment.

Second, monitoring procedures may be systematically implemented before and after training sessions. Pre-training well-being assessment may assist coaches in adjusting training loads according to the athletes’ physical and mental state, whereas post-session sRPE may provide a practical estimate of internal load. In this context, CMJ assessment may serve as one indicator of neuromuscular readiness within a broader monitoring framework.

Third, training organization based on a structured microcycle model, including retraining and competition microcycles (one-match and two-match formats), may represent a practical and flexible approach to periodization in semi-professional female handball. Retraining microcycles may allow relatively greater physical and technical loading, whereas competition microcycles may emphasize tactical and integral preparation according to match density.

Fourth, coaches may consider prioritizing tactical and integral training during pre-match periods, as these components demonstrated the strongest relationships with match outcomes in the investigated team.

Finally, practitioners may benefit from adopting a systems-oriented approach that integrates training load, athlete well-being, physical performance, and match analysis into a unified decision-making framework, potentially supporting performance management throughout the competitive season.

### 4.5 Conclusions

The present study suggests that performance in semi-professional female handball players is influenced by the integrated interaction of training load, athlete mental state, physical performance, and match-specific factors. Match success appeared to be primarily associated with defensive performance variables, particularly first-half defensive efficiency and goalkeeper effectiveness.

Training load monitoring based on sRPE-derived metrics (TWL, monotony, strain, and A:CWR) revealed systematic variations across the season, reflecting the demands of different microcycle structures. Despite these fluctuations, physical performance indicators, including endurance, strength, and throwing velocity, were generally maintained or improved without a traditional preparatory period.

Importantly, athlete preparedness appeared to be effectively developed through a structured microcycle model consisting of retraining and competition microcycles in one- and two-match formats. This model may allow dynamic load regulation and targeted adaptation according to competitive demands.

Overall, these findings support the potential applicability of flexible, monitoring-informed periodization in semi-professional female handball. The results may indicate that structured microcycle planning represents a practically relevant alternative to more rigid traditional models within specific team and competitive contexts. Performance in team handball should therefore be understood as a complex and multidimensional system in which training organization, monitoring variables, and tactical execution are closely interrelated.

### 4.6 Limitations

The present study provides an applied framework for integrating training and monitoring processes throughout a handball competitive season.

First, coaches may use the reported reference values of internal load (session duration × sRPE), total weekly load (TWL), monotony, strain, and A:CWR as contextual benchmarks when planning and evaluating training programs. However, these values should be interpreted relative to the specific characteristics of the athlete group and competitive environment.

Second, monitoring procedures may be systematically implemented before and after training sessions. Pre-training well-being assessment may assist coaches in adjusting training loads according to the athletes’ physical and mental state, whereas post-session sRPE may provide a practical estimate of internal load. In this context, CMJ assessment may serve as one indicator of neuromuscular readiness within a broader monitoring framework.

Third, training organization based on a structured microcycle model, including retraining and competition microcycles (one-match and two-match formats), may represent a practical and flexible approach to periodization in semi-professional female handball. Retraining microcycles may allow relatively greater physical and technical loading, whereas competition microcycles may emphasize tactical and integral preparation according to match density.

Fourth, coaches may consider prioritizing tactical and integral training during pre-match periods, as these components demonstrated the strongest relationships with match outcomes in the investigated team.

Finally, practitioners may benefit from adopting a systems-oriented approach that integrates training load, athlete well-being, physical performance, and match analysis into a unified decision-making framework, potentially supporting performance management throughout the competitive season.

## Data Availability

The original contributions presented in the study are included in the article/Supplementary Material, further inquiries can be directed to the corresponding author.
